# Nodes with the highest control power play an important role at the final level of cooperation in directed networks

**DOI:** 10.1038/s41598-021-93144-5

**Published:** 2021-07-01

**Authors:** Ali Ebrahimi, Marzieh Yousefi, Farhad Shahbazi, Mohammad Ali Sheikh Beig Goharrizi, Ali Masoudi-Nejad

**Affiliations:** 1 Laboratory of Systems Biology and Bioinformatics (LBB), Institute of Biochemistry and Biophysics, University of Tehran, Tehran, Iran; 2 Department of Physics, Isfahan University of Technology (IUT), Isfahan, Iran; 3 Atherosclerosis Research Center, Baqiyatallah University of Medical Sciences, Tehran, Iran

**Keywords:** Computational models, Network topology, Statistical methods

## Abstract

Controllability of complex networks aims to seek the lowest number of nodes (the driver nodes) that can control all the nodes by receiving the input signals. The concept of control centrality is used to determine the power of each node to control the network. The more a node controls the nodes through connections in the network, the more it has the power to control. Although the cooperative and free-rider strategies and the final level of cooperation in a population are considered and studied in the public goods game. However, it is yet to determine a solution to indicate the effectiveness of each member in changing the strategies of the other members. In a network, the choice of nodes effective in changing the other nodes’ strategies, as free-riders, will lead to lower cooperation and vice versa. This paper uses simulated and real networks to investigate that the nodes with the highest control power are more effective than the hubs, local, and random nodes in changing the strategies of the other nodes and the final level of cooperation. Results indicate that the nodes with the highest control power as free-riders, compared to the other sets being under consideration, can lead to a lower level of cooperation and are, therefore, more effective in changing the strategies of the other nodes. The obtained results can be considered in the treatment of cancer. So that, destroying the tumoral cells with the highest control power should be a priority as these cells have a higher capability to change the strategies of the other cells from cooperators to free-riders (healthy to tumoral).

## Introduction

To model the complex systems by using the networks, the system agents are considered as nodes and their relationship as edges^1–5^. An analysis of a network is done in two ways: static and dynamic.

In a static analysis, the time-independent topologic properties of a network are calculated. These topologic properties are classified into two groups: global and individual. Distribution of the node degrees, the path length, and the network diameter are the examples of global topologic properties. These properties specify how the entire system behaves and acts as a whole. Individual topologic features indicate each network element's importance as nodes, edges, motifs, modules and etc. Power and centrality measures such as closeness, betweenness, bridging, and control centrality are examples of these properties; each of the above-mentioned examples indicates a difference in specificity and the importance of the nodes in maintaining the network stability.

Unlike a static analysis, in a dynamic analysis, the focus is on the temporal evolution of the network’s components. What is essential in analyzing the dynamics of the networks is the collective behavior of agents living in them^6^. There are various theoretical methods for improving the synchronous movement performance in the dynamic networks. Changing a node’s relationship structure in the network, increasing the weight of links between the nodes, adding or removing edges to or from the network are the examples of the most commonly used methods^7^. However, due to some limitations, most of the above-mentioned methods cannot be implemented in a network. An efficient method for improving the synchronous movement's performance on the networks is the external control of the nodes.

A dynamical system will be controllable when a proper choice of inputs can transfer the system from any initial state to the desired one through a finite number of the steps^8–10^. The main problem in controllability is finding the lowest number of the nodes in the networks which enables the control of all the nodes when affected by the input signals. Targeted controllability searches for the lowest number of the nodes in the networks which can control a particular target.

To find the driver nodes in the directed networks, the minimum input theorem, which is based on the Lin's structural controllability theorem^11^, indicates the relationship between the structural controllability and the maximum matching^12^. Moreover, to implement a target control, a greedy algorithm is applied based on the multiple maximal matching^13^. Target controllability is an NP-hard problem^14^; it means that this is at least, as hard as the hardest problems in NP-problem (Non-deterministic Polynomial-time) problem. and many algorithms have been developed to improve its outcomes^15–20^. In target controllability, the placement of the non-target nodes in the control signals' pathway can have some side effects on many applications. Minimizing the intermediate nodes between the driver nodes and the target nodes is among the most challenging controllability problems. To this end, we use an algorithm which is based on the path length between each pair of nodes in a directed network. In this algorithm, the highest number of the target nodes controlled by each node is given based on the least intermediate nodes between the driver and target nodes^19^.

Control centrality is an individual topologic characteristic in a network used to determine each node's power to control the network^21^. In other words, the more a node can control the other nodes via connections in the network, the higher it is ranked in control centrality and vice versa. The importance of control and the significance of the implemented applications to find the driver nodes have been subject to great attentions^22–29^.

The public goods game is a group game inspecting the collaboration among the members of a population through holding competition among the different groups. Colleague (cooperator) and free-rider (defector) are the two existing strategies randomly distributed through the population^30^. Unlike the free-riders (*D*), Collaborators (*C*) pay cost for collaboration. The exact situation which is observed in societies, in which just some pay tax, but everyone can reap all the benefits.

A game such as the public goods game can have more than two strategies, such as punishers and rewarders. Punishers can encourage the defectors that they cooperate with others by using costs and sanctions. Also, rewarders can promote cooperation by paying rewards to the cooperators. The results demonstrate that this method can change the cooperation levels considerably. However, it does not mean that the punishers or rewarders can be the main winners, but they can modify the cooperation levels and coexist with other strategies^31–33^. In the other work, the authors have proposed social exclusion as an idea for promoting cooperation by using two concepts: prosocial pool exclusion and antisocial pool exclusion. The replicator equation has been used in this research to investigate the cooperation level in a well-mixed population^34^.

Also shown that exclusion positively affects cooperation regardless of dilemma type^35^. The fraction of cooperators in the N-person snowdrift game demonstrated more amounts by using the pool-rewarding concept. The well-mixed population has been the goal of this research^36^.

Many kinds of research have already been about spatial games. Also, the different configuration of networks has been an essential subject in various works. According to the results, spatial games can give rise to the coexistence of different strategies. For example, this result had been taken about Prisoner's Dilemma (PD) problem, individually. It seems that the coexistence level can depend on competition (update) rules and the symmetric or asymmetric configuration of cells in a network^37,38^.

Some works have used a non-linear benefit function in a public goods game to investigate the level of cooperation. The sigmoid function is a well-known function that considers in such researches. The results showed that the number of cooperator strategies could have a middle value between *0* and *1*. Also, the authors showed that the cost-to-benefit ratio could affect the number of cooperators^39^. In the other research, it has shown that the moods of persons can influence the game results. The authors have considered positive, negative, and neutral perspectives and investigated the final results. They illustrated that, in general, the hostile persons could extinct if the tendency of the neutral persons was positive mood persons, and so, the cooperation can increase quickly^40^.

In this manuscript, we show that the nodes with the highest control power play a key role in the outcomes of a public goods game. Results indicate that, they will more strongly direct the population towards betrayal in case they are selected as the free-riders.

## Methods

Consider a directed network *G(A)* with *N* nodes, whose dynamics are given by the following linear differential equation:

1 $$dx\left( t \right)/d\left( t \right) = Ax\left( t \right) + Bu\left( t \right)$$

In which $A_{{N \times N}}$ is the connectivity matrix between the nodes. The $x\left( t \right) = \left( {x_{1} \left( t \right), \ldots ,x_{N} \left( t \right)} \right)^{T}$ vector determines the state of the $N$ nodes at the moment *t*. $B_{{N \times M}} \left( {M \le N} \right)$ indicates the driver nodes, in which the elements in its columns are zero except for those which correspond to the driver nodes, which are nonzero. The network is controlled by a time-dependent incoming vector $u\left( t \right) = \left( {u_{1} \left( t \right), \ldots ,u_{M} \left( t \right)} \right)$^10,41^.

The main problem in controllability is finding a proper subset of the network nodes with the lowest cardinality in such a way that when they undergo the input signals, they put all the network nodes in the desired state. These nodes are called the driver nodes.

Based on the Kalman condition^8^, the network will be controlled via the nodes related to the *B* matrix, if $Q_{{N \times NM}} = \left[ {B\left| {AB} \right|A^{2} B\left| \ldots \right|A^{{N - 1}} B} \right]$ has a complete rank, i.e., $rank\left( Q \right) = N$.

The controllability method is used to determine the number of the nodes which are under the control of each node. The method is based on the path lengths between each pair of nodes. In this method, for every node in the network, the maximum number of the nodes which are under the control will be achieved provided that, the number of the intermediate nodes is minimized^19^. The nodes with the highest control centrality (HCC) are then detected and considered as the set of nodes which initially employ a free-rider strategy. To assess the performance of the nodes with the highest control power, we make a comparison between them and the other three groups of the nodes employing the free-rider strategy:

1. Nodes with the highest out-degree,
2. Local selection of nodes, in which, first, a node is randomly selected, and then, its first, second and … *k*th neighbors are, hierarchically will be chosen.
3. Random selection of nodes, in which every node has an equal probability to be selected as a free-rider.

To start the public goods game, one selects a member of the population as the first player and then considers a group of nodes with a specific size, centered around that member, as its co-players.

In the public goods game with a linear benefit function, the payoff of a defector in the groups is given by:

2 $$P_{D} = \frac{{r^{*} j^{*} c}}{n}$$

and the payoff of a cooperator is given by:

3 $$P_{c} = P_{D} - c$$

In which *r* is the synergic factor, *j* is the number of cooperators in a group, *c* is the cost paid for the beginning of cooperation and *n* is the group size. In the simplest variant, *n* is the number of the first neighbors plus the player itself.

At the next step of the game, we randomly select one of the first player's nearest neighbors and calculate its benefit (the second player). Finally, based on the benefits calculated and considering the selective update rule, decisions will be made about changing the first player's strategy.

The Eqs. (2) and (3) are the linear payoffs commonly used in the standard public goods game. However, in reality, like biology, the nonlinear benefit functions seem to be superior. In some biological processes such as synthesizing a specific enzyme, secretion will stop when a certain enzyme concentration threshold is achieved. It means that enzyme secretion depends on the enzyme concentration in the medium. Generally, most biological processes have a threshold and a saturation limit; hence the benefit function corresponding to these processes is to a large extent close to a sigmoid function. It is shown that unlike a linear function, using a nonlinear benefit function gives rise to completely different outcomes^42^. We have used a non-linear benefit function that is different from the traditional public goods game. Because the biological systems often show non-linear behavior. So, it seems this is a logical idea that the non-linear behavior has been considered instead of a linear function and the competition between cooperators and defectors is based on a non-linear benefit.

In the present paper, a sigmoid function is used to calculate the benefit function:

4 $$b\left( j \right) = \frac{{l\left( j \right) - l\left( 0 \right)}}{{l\left( n \right) - l\left( 0 \right)}}$$

In which:

5 $$l\left( j \right) = \frac{1}{{1 + exp\left( {s\left( {h - j{\text{/}}n} \right)} \right)}}$$

In Eqs. (4) and (5); *j* which is the number of cooperators in a group, is determined by the diffusion length *d*^43^. *h* and *s* are the turning point and the slope of the payoff function, respectively, and *n* is the group size.

The benefit functions of a cooperator and a defector in a group containing *j* cooperators are given by:

6 $$P_{D} = b\left( j \right)\quad \& \quad P_{C} = P_{D} - c$$

We use both the deterministic and the stochastic update rules for updating the first player’s strategy. Under the deterministic update rule, the first player will select its neighboring co-player’s strategy with a probability of *1* if its neighbor gets more payoffs; otherwise, it will retain its own strategy. However, under the stochastic (Glauber) update rule, the following probability will be used for switching the first player’s strategy:

7 $$p\left( {x \to y} \right) = \frac{1}{{1 + exp\;(\beta \left( {P_{x} - P_{y} } \right)}}$$

where $P_{x}$ and $P_{y}$ are the first and the second players’ benefits, respectively, and *β* controls the level of uncertainty (the large *β* corresponds to a low level of noise and vice versa).

## Results

### Synthesized networks

First, we investigate the public goods game in the directed scale-free networks as well as a toy network. The Barabasi-Albert (BA) preferential attachment algorithm^44^ is employed to generate the directed scale-free networks with *400* nodes in two structures: sparse (< *k*> = *10* ) and dense (< *k*>= *20*) *.* Then, *20* percent of the network nodes (*80* nodes) are selected as the initial free-riders. Four groups of nodes are chosen as the initial defectors: (1) nodes with the highest control centrality (HCC), (2) nodes with the maximum out-degree, (3) local nodes, and 4) randomly selected nodes. Considering the networks' directional structure, each player can affect only the neighbors' strategies towards which there is a directed connection toward them.

**Figure 1:**
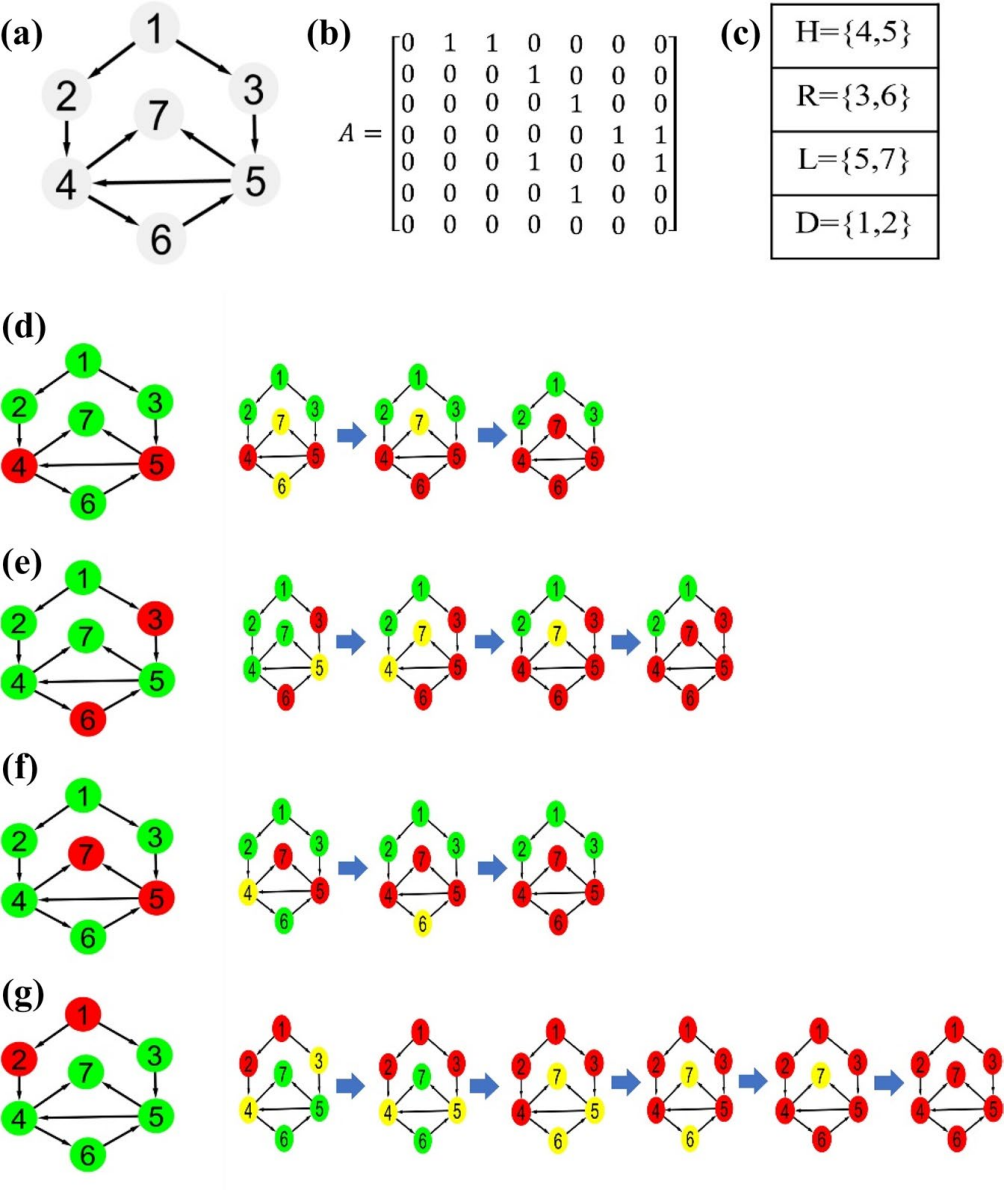
The effect of an initial choice of free-rider nodes on strategy switching by the other network nodes. (**a**) A directed network with *7* nodes and *9* edges. (**b**) The Network adjacency matrix. (**c**) H: the nodes with the maximum out-degree, R: random selection of the initial target nodes, L: local selection of the nodes, D: the nodes with the highest control power in the network. (**d**) The selection of the hub nodes as the initial target for the free-rider nodes and an analysis of the changes in the strategies of the other nodes, where the final level of cooperation amounts to $${\raise0.7ex\hbox{$3$} \!\mathord{\left/ {\vphantom {3 7}}\right.\kern-\nulldelimiterspace} \!\lower0.7ex\hbox{$7$}}$$. (At each stage, the free-rider nodes are displayed in red, their immediate neighbors are displayed in yellow and their cooperating nodes are displayed in green). (**e**) The final level of cooperation through a random selection of the free-rider nodes amounts to $${\raise0.7ex\hbox{$2$} \!\mathord{\left/ {\vphantom {2 7}}\right.\kern-\nulldelimiterspace} \!\lower0.7ex\hbox{$7$}}$$. (**f**) By selecting the local nodes as the free-riders, the final level of cooperation will be $${\raise0.7ex\hbox{$3$} \!\mathord{\left/ {\vphantom {3 7}}\right.\kern-\nulldelimiterspace} \!\lower0.7ex\hbox{$7$}}$$. (**g**) The selection of the nodes with the highest control power as the free-riders results in a hierarchical strategy change in a network’s total nodes and as a result, the network’s complete betrayal.

Figure 1 represents a toy network, where a directed network with *7* nodes and *9* edges is considered. In each of the aforementioned four condition sets, the first two nodes have the *D* strategy; these two nodes’ impact on the other nodes’ strategy is presented step by step. As visible in the picture, selecting the two nodes with the highest control centrality in the network as free-riders results in the network’s movement towards a complete betrayal and zero cooperation.

**Figure 2:**
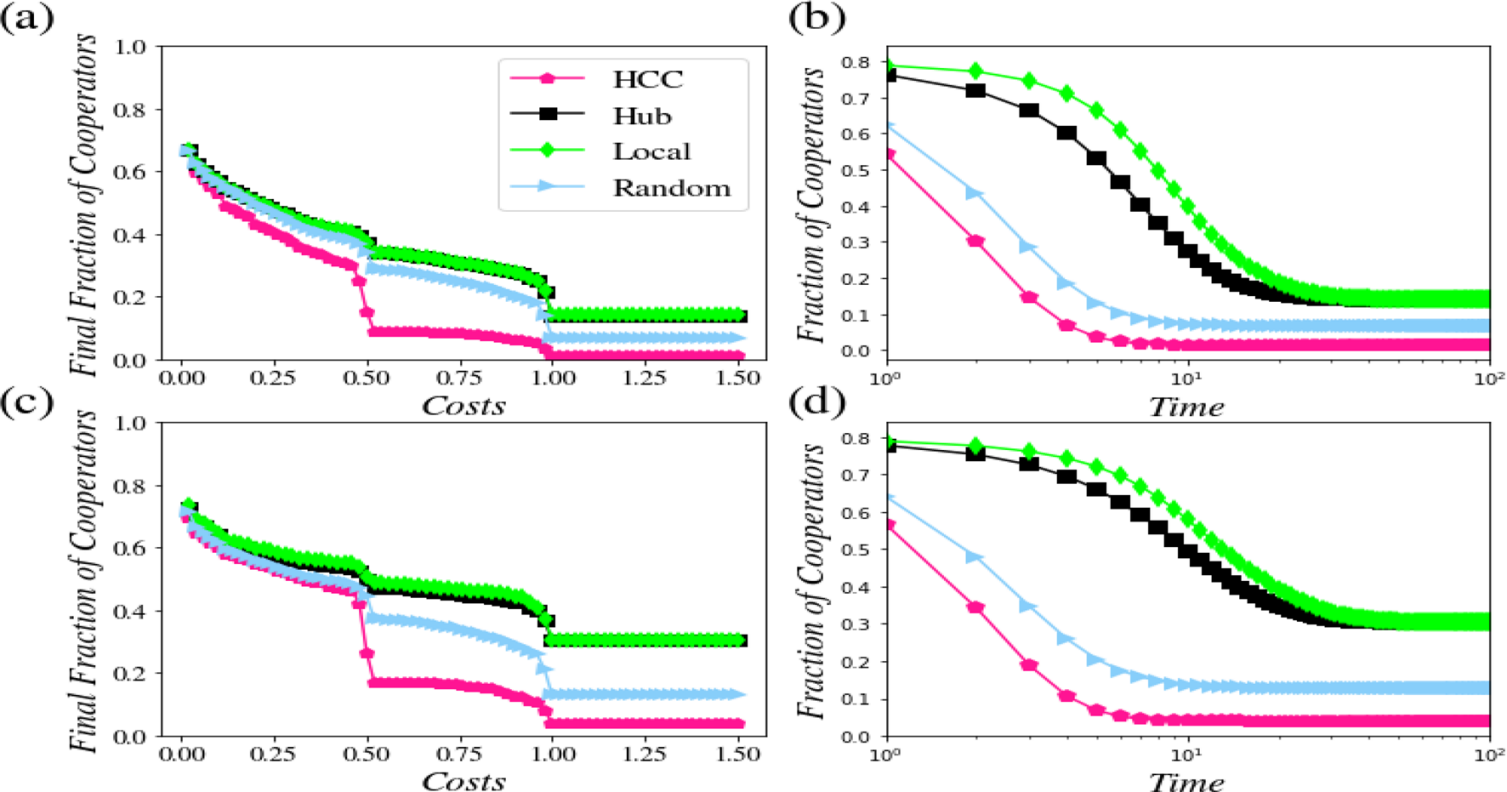
In dense and sparse networks with 400 nodes: an investigation into the effect of an initial choice of the free-rider nodes on controlling the scale-free networks through a deterministic update. (**a**) An analysis of the cooperation level in a dense network: An increase in the cooperation cost will lead to a decrease in the amount of cooperation. Under similar conditions, the nodes with the highest control ability, lead to a lower level of cooperation. (**b**) An analysis of the dense network’s dynamics: if the driver nodes are selected as the free-riders, the cooperation level will decrease (*c* = *1*). (**c**) An analysis of the cooperation level in a sparse network: In random selection and nodes with the highest control power, compared by selecting hub and local nodes, will lead to a higher level of cooperation. (**d**) An analysis of the dynamics of a sparse network: just like a dense network, the velocity of achieving a steady state is higher, where the nodes with the highest control power are selected (*c* = *1*).

In Fig. 2, parts b and d display the results related to the dynamic behavior of the dense and sparse networks when the deterministic updating rule is applied. This figure shows that the edge density of the network has a remarkable effect on cooperation. Based on the results, the level of cooperation is on average *20*% higher in the sparse network.

The cooperation cost (*c*) is an important parameter in the public goods game. A rise in the costs will lead to a reluctance of people to cooperate, which in turn can result in the stoppage of the cooperation at some point. However, the generated outcomes show that there are cost intervals, at which the level of cooperation shows a plateau. Such an effect can be explained with regard to the network directionality, as in the case of a directed network, in which one node will be affected by the other one, if only there exists a directed link from the second node to the first one.

In Fig. 2, parts a and c display the final cooperation results yielded in the dense and sparse networks. A comparison drawn between these two networks shows that the denser a network is, the less it becomes cooperative. Indeed, controlling the network towards betrayal is easier if the network is denser. The above conclusions are all based on Monte-Carlo calculations, and averaged out of *20* independent configurations.

**Figure 3:**
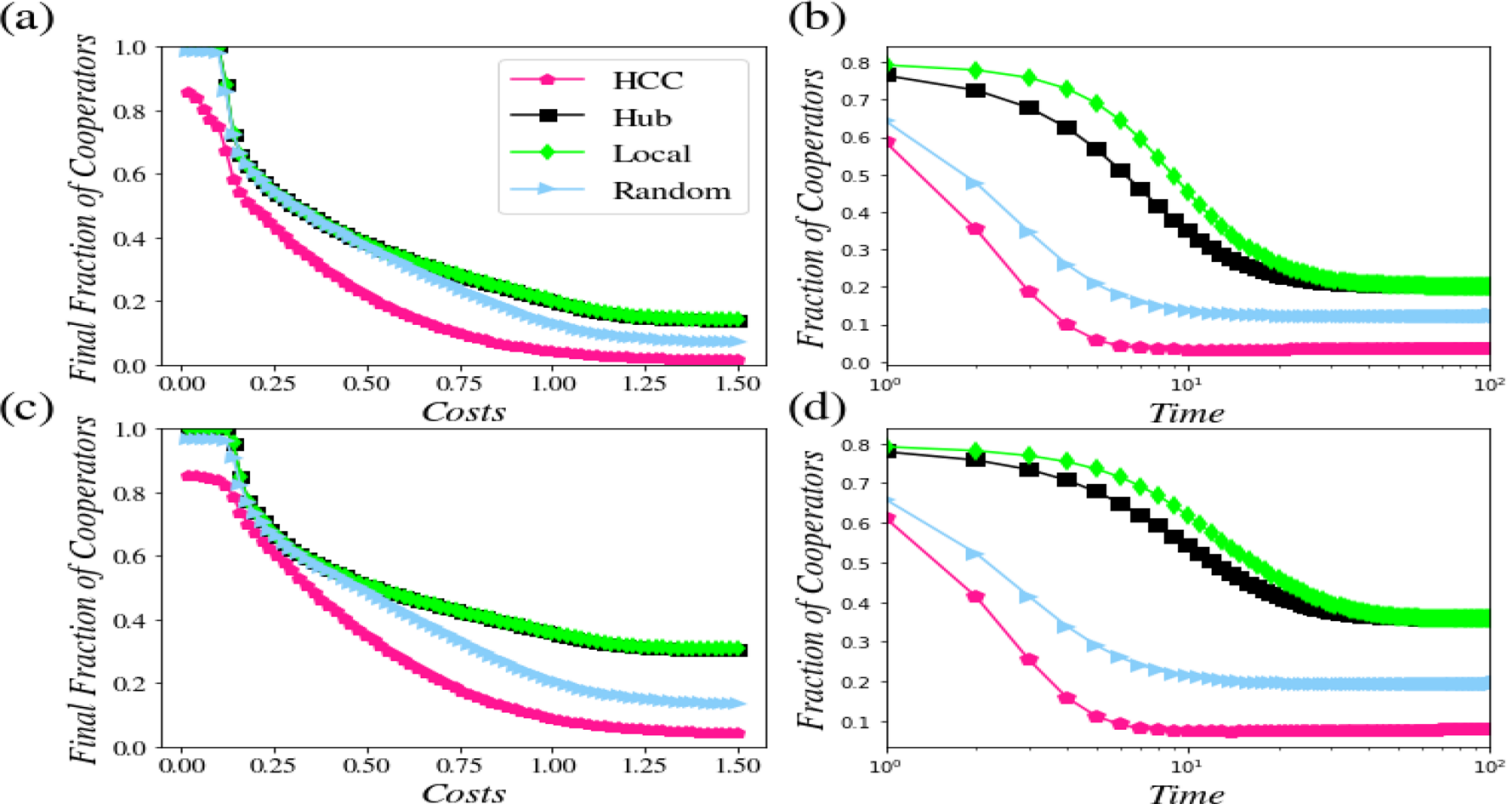
An analysis of the effect of the initial node selection on the network control using the Glauber update rule. The other conditions are as the ones shown in Fig. 2.

Figure 3 displays the effect of the Glauber update rule on the level of cooperation in networks. In dense networks, the effect of the predominance of nodes with the highest control centrality will be easy to observe under the Glauber random update. The results obtained for the other three choices of the free-rider nodes will be the same as long as *c* = *0.6*. In the case of *c* > *0.6*, however, the effectiveness of the random choice can be very well observed. Moreover, the results won’t vary in case the hub and local nodes are selected as the initial targets of the free-rider nodes. As a general outcome, we can say that in comparison to sparse networks, in dense networks, there is a higher chance of betrayal regardless of the kind of the update rule applied.

### Real networks

**Figure 4:**
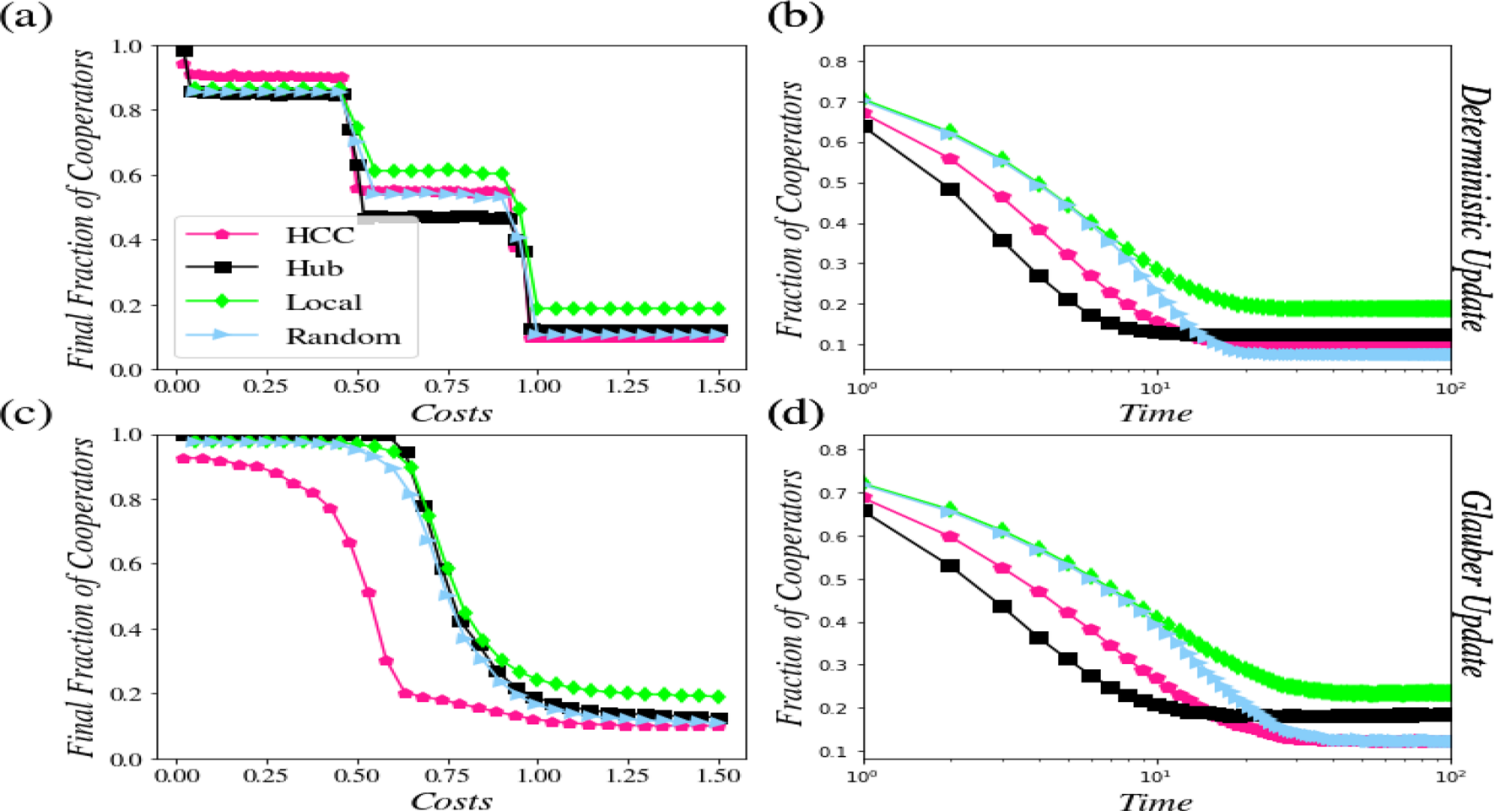
Results of the *s420* network. (**a**) An analysis of the cooperation level based on the deterministic update rule. (**b**) The network dynamics in achieving a steady state under the deterministic update rule (*c* = *1*). (**c**) An analysis of the cooperation level based on the Glauber update rule. (**d**) The network dynamics in achieving a steady state under the Glauber update rule (*c* = *1*).

In this section, we perform the public goods game in the real networks. We use the three gold-standard directed networks: *s420* with *252* nodes and *399* edges^45^, *s208* with *122* nodes and *189* edges^45^, and *Mangrove* with *97* nodes and *1492* edges^46^. The results are displayed in Figs. 4, S1, and S2, respectively.

The outcomes presented in Fig. 4 demonstrate that in *s420* network under deterministic conditions, transitions occur, similar to what we found in the synthesized networks. Furthermore, under deterministic conditions, the significance of the nodes with the highest control centrality in changing the strategies of the other nodes is clear as long as *c* > *1*. However, under the Glauber update rule, the role of the nodes with the highest control centrality can be well observed at all the cost values. It can be seen that the effect of hubs is considerable to reach the steady-state sooner in the network dynamics.

The results of the *s208* network, presented in Fig. S1, indicate that in the deterministic state, the selection of the nodes with the highest control power as the initial defectors in the network has a moderate effect on the propagation of betrayal in the network at the intermediate values of the cost. This is true, despite the fact that under the Glauber updating rule, these nodes, compared to the other nodes, will better drive the network towards stabilizing the *D* strategy. Figure S1b and d show that under both the deterministic and Glauber updating rules, reaching a steady-state takes longer when the nodes with the highest control centrality are selected as the initial defectors.

Figure S2 presents the results of the *Mangrove* network under the two update rules. The results shown in this figure indicate that under both of the updating rules, the selection of the four groups as the initial defectors leads to a clear distinction in the final level of cooperation at all the cost values.

## Conclusion

Cooperation is of critical importance to a society’s development and enrichment. The way the members of a population cooperate with or betray each other can affect the outcomes of the society-related processes. In such a condition, what matters is a strategy change among the members of a society who participate in this process. Efforts to change and have an effect on one’s mind and to be placed among the free-riders deserve great attention. For example, in a poll, changing the voters’ strategies leads to a change in the election outcomes.

In this paper, we implemented the concept of controllability in the public goods game to control the level of cooperation in a population residing in a network. The results obtained in both the synthesized and real networks indicate that implementing the defector strategy in a set of nodes with a higher control power is the most effective way to change the strategies of the other nodes from cooperation to betrayal.

Since in a directed network, with any specified path length, each node can control up to a maximum of one other node, this conclusion is also theoretically valid. Therefore, for a set of nodes that is under the control of each node, there must be paths with different lengths from that node is available. As a result, there are often many paths, with different lengths, to other nodes of the network from the nodes which have high control power. Consequently, these nodes can affect the network’s other nodes in different steps, and change their strategy from a cooperator to a defector.

As an application for treating cancer, in the early stages of cancer, the healthy cells of a cancerous tissue are considered as cooperating members and its tumoral cells as free-riding members. The link which is formed between any two cells through signaling between them defines the complex network corresponding to the inter-cellular communication. The topology of this assembled web determines the control power of each node. Based on the results obtained in this paper, and considering the existing network between the cells of the target tissue, tumoral cells with high control power, unlike the other tumoral cells in the tissue, are more capable of tumorizing healthy cells (changing the strategy of cooperating members to free riders’ strategy) and advancing the cancer process (directing the network toward complete betrayal). Therefore, destroying tumoral cells with high control power, compared to the destruction of other tumoral cells in the tissue, is more effective in preventing the progression of cancer and tumorization of tissue’s healthy cells.

Moreover, the presented results demonstrate that the outcomes of the two Glauber and deterministic updating rules are so different. The network shows more resistance to the *D* steady state’s achievement when the Glauber rule is applied. Sparsity or density can also play a role in determining the amount of cooperation. Regardless of the mimicking rule, in a denser network, conditions are more suitable for propagating the *D* strategy in the network. Therefore, applying the deterministic updating rule and using the dense networks can result in the highest inclination towards the *D* strategy. These results also are consistent with the fact that the existence of cavities on networks facilitates cooperation^47–49^.

## Supplementary information

Supplementary Information.
