# Supplementary material for: Nodes with the highest control power play an important role at the final level of cooperation in directed networks
Source: Sci Rep. 2021 Jul 1;11:13668. doi: 10.1038/s41598-021-93144-5 (PMC8249622; doi:10.1038/s41598-021-93144-5)
Supplement: Supplementary file 1 — Supplementary Information. [file 41598_2021_93144_MOESM1_ESM.docx]

**Supplementary Material**

**Nodes with the highest control power play an important role at the final level of cooperation in directed networks**

**Ali Ebrahimi^1^, Marzieh Yousefi^2^, Farhad Shahbazi^2^, Mohammad Ali Sheikh Beig Goharrizi^3^, Ali Masoudi-Nejad^1*^**

1. Laboratory of Systems Biology and Bioinformatics (LBB), Institute of Biochemistry and Biophysics, University of Tehran, Tehran, Iran
2. Department of physics, Isfahan University of Technology (IUT), Isfahan, Iran
3. Atherosclerosis research center, Baqiyatallah University of Medical Sciences, Tehran, Iran

***Corresponding Author**

Ali Masoudi-Nejad, Ph.D,

Laboratory of Systems Biology and Bioinformatics (LBB)

Institute of Biochemistry and Biophysics

University of Tehran, Tehran, Iran.

E-mail: [amasoudin@ut.ac.ir](mailto:amasoudin@ut.ac.ir)

WWW: <http://LBB.ut.ac.ir>

**
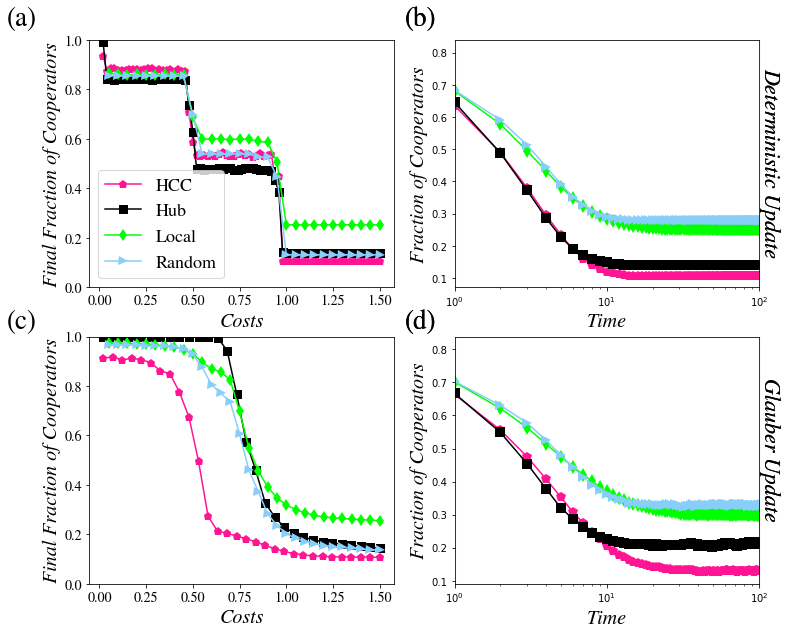
**

**Fig S1. Results of the s208 network** **a)** An analysis of the cooperation level based on the deterministic update rule. **b)** The network dynamics in achieving a steady state under the deterministic update rule (c=1). **c)** An analysis of cooperation level based on the Glauber update rule. **d)** The network dynamics in achieving steady state under the Glauber update rule (c=1).

**
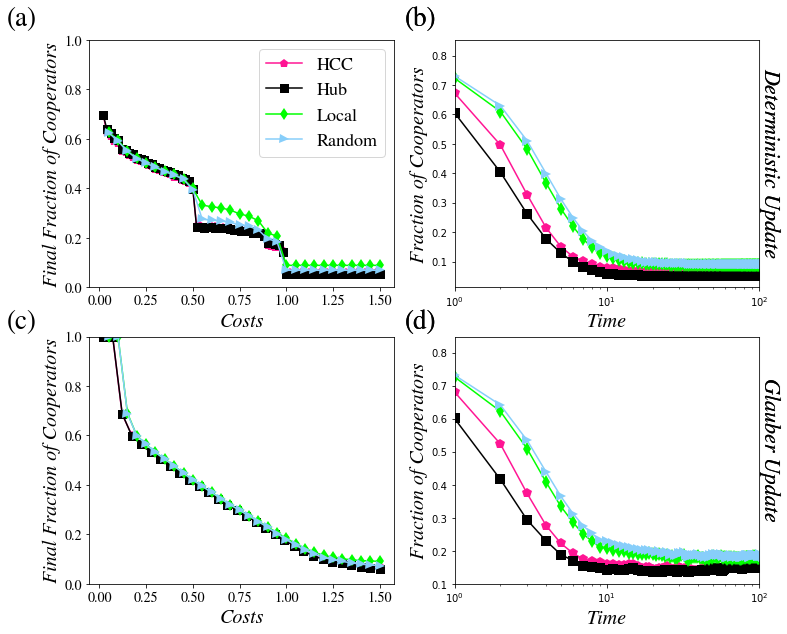
**

**Fig S2.** **Results of the Mangrove network** Designations are as Fig S1.
